# A weak instrument F-test in linear IV models with multiple endogenous variables^☆^

**DOI:** 10.1016/j.jeconom.2015.06.004

**Published:** 2016-02

**Authors:** Eleanor Sanderson, Frank Windmeijer

**Affiliations:** a Department of Economics, University of Bristol, UK; b CMPO and IEU, University of Bristol, UK; c CEMMAP/IFS, London, UK

**Keywords:** Weak instruments, Multiple endogenous variables, F-test

## Abstract

We consider testing for weak instruments in a model with multiple endogenous variables. Unlike Stock and Yogo (2005), who considered a weak instruments problem where the rank of the matrix of reduced form parameters is near zero, here we consider a weak instruments problem of a near rank reduction of one in the matrix of reduced form parameters. For example, in a two-variable model, we consider weak instrument asymptotics of the form $\pi_{1}=\delta \pi_{2}+c/\sqrt{n}$ where $\pi_{1}$ and $\pi_{2}$ are the parameters in the two reduced-form equations, c is a vector of constants and n is the sample size. We investigate the use of a conditional first-stage F-statistic along the lines of the proposal by Angrist and Pischke (2009) and show that, unless δ=0, the variance in the denominator of their F-statistic needs to be adjusted in order to get a correct asymptotic distribution when testing the hypothesis $H_{0}:\pi_{1}=\delta \pi_{2}$. We show that a corrected conditional F-statistic is equivalent to the Cragg and Donald (1993) minimum eigenvalue rank test statistic, and is informative about the maximum total relative bias of the 2SLS estimator and the Wald tests size distortions. When δ=0 in the two-variable model, or when there are more than two endogenous variables, further information over and above the Cragg–Donald statistic can be obtained about the nature of the weak instrument problem by computing the conditional first-stage F-statistics.

## 1 Introduction

Following the work of Staiger and Stock (1997) and Stock and Yogo (2005), testing for weak instruments is now commonplace. For a single endogenous variable model, the standard first-stage F-statistic can be used to test for weakness of instruments, where weakness is expressed in terms of the size of the bias of the IV estimator relative to that of the OLS estimator, or in terms of the magnitude of the size distortion of the Wald test for parameter hypotheses. Stock and Yogo (2005) tabulated critical values for the standard F-statistic that have been incorporated in software packages.

For multiple endogenous variables, inspection of the individual first-stage F-statistics is no longer sufficient. The Cragg and Donald (1993) statistic can be used to evaluate the overall strength of the instruments in this case, and Stock and Yogo (2005) have tabulated critical values of the minimum eigenvalue of the Cragg–Donald statistic for testing weakness of instruments. They derive the limiting distributions under weak instrument asymptotics where the reduced form parameters are local to zero in each reduced form equation, and obtain critical values that are conservative in the sense that they are rejecting the null of weak instruments too infrequently when the null is true.

In this paper, we are interested in analysing tests for weak instruments in a model with multiple endogenous variables in a setting where the reduced form parameters are not local to zero, but where the reduced form parameter matrix is local to a rank reduction of one. In this case, the values of the F-statistics in each of the first-stage equations can be high, but the identification of (some of) the model parameters is weak. We will focus initially on a model with two endogenous variables. The weak instrument asymptotics we consider are local to a rank reduction of one, of the form

$$\pi_{1}=\delta \pi_{2}+c/\sqrt{n}\text{,}$$

where $\pi_{1}$ and $\pi_{2}$ are the parameters in the two reduced-form equations, c is a vector of constants and n is the sample size. We call these asymptotics LRR1 weak instrument asymptotics.

We will focus solely on the properties of the 2SLS estimator. We investigate the use of a conditional first-stage F-statistic along the lines of the proposal by Angrist and Pischke (2009) and show that the variance formula in the denominator of their F-statistic needs to be adjusted in order to get a correct asymptotic distribution when testing the null hypothesis, in the two-variable model, $H_{0}:\pi_{1}=\delta \pi_{2}$. We further show that the resulting new conditional F-statistic is equivalent to the Cragg–Donald minimum eigenvalue statistic. Using our weak instrument asymptotics we show that this conditional F-statistic cannot be used in the same way as the Stock and Yogo (2005) procedure for a single endogenous variable to assess the magnitude of the relative bias of the 2SLS estimator of an individual structural parameter. This is because the OLS bias expression contains additional terms such that the expression for the bias of the 2SLS estimator relative to that of the OLS estimator does not have the simple expression as in the one-variable case. However, the total relative bias can be bounded as can the size distortions of Wald tests on the structural parameters.

In a two-endogenous-variable model the conditional F-statistics for each reduced form are equivalent to each other and to the Cragg–Donald minimum eigenvalue statistic under our LRR1 weak instrument asymptotics. This holds unless δ=0, in which case the local rank reduction is due to the fact that $\pi_{1}$ is local to zero and the first-stage F-statistic for $x_{1}$ will be small and that for $x_{2}$ will be large. In this case, both the Angrist–Pischke F-statistic and our conditional F-statistic for $x_{1}$ can be assessed against the Stock–Yogo critical value, and the 2SLS estimator for the structural parameter on $x_{2}$ is consistent. Additional information can also be obtained from our conditional F-statistics when there are more than two endogenous variables, as they will identify which variables cause the near rank reduction. For example, if in a three variable model the near rank reduction is due to the reduced form parameters on two variables only, the conditional F-statistic for the third variable will remain large giving the researcher valuable information about the nature of the problem and directions for solving it. We also show that the 2SLS estimator for the structural parameter of the third variable is consistent in that case.

The paper is organised as follows. In Section  2 we introduce the linear model with one endogenous variable and summarise the Staiger and Stock (1997) and Stock and Yogo (2005) results for testing for weak instruments. Section  3 considers weak instrument test statistics for the linear model with two endogenous explanatory variables and introduces the new conditional F-tests. Section  4 considers the relative bias and Wald test size distortions for the 2SLS estimator under the LRR1 weak instrument asymptotics as outlined above and presents some Monte Carlo results for the two-variable model. Section  4 also shows the usefulness of the conditional F-test statistics in a model with more than two endogenous variables. Finally, Section  5 concludes.

## 2 Weak instrument asymptotics in one-variable model

In this section we follow the basic Staiger and Stock (1997) and Stock and Yogo (2005) setup. The developments of the weak instrument setup and concepts for the one-variable model play an important role when we expand the model to multiple endogenous variables in the next section. The simple model is

(1) y=xβ+u,

where y, x, and u are n×1 vectors, with n the number of observations. There is endogeneity, such that E(u|x)≠0. The reduced form for x is

(2) x=Zπ+v,

where Z is a $n\times k_{z}$ matrix of instruments and v is n×1. For individual $u_{i}$ and $v_{i}$ we assume,

$$\left(\begin{array}{c} u_{i}\\ v_{i} \end{array}\right)∼\left(0,\Sigma \right)\text{;}\;\Sigma =\left(\begin{array}{cc} \sigma_{u}^{2} & \sigma_{uv}\\ \sigma_{uv} & \sigma_{v}^{2} \end{array}\right)\text{.}$$

The 2SLS estimator is given by

β^2SLS=x′PZyx′PZx=β0+x′PZux′PZx,

where $P_{Z}=Z{\left(Z^{\prime }Z\right)}^{-1}Z^{\prime }$.

The concentration parameter is given by

$$CP=\frac{\pi^{\prime }Z^{\prime }Z\pi }{\sigma_{v}^{2}}$$

and is a measure of the strength of the instruments, see Rothenberg (1984). A small concentration parameter is associated with a bias of the 2SLS estimator and deviations from its asymptotic normal distribution.

A simple test whether the instruments are related to x is of course a Wald or F-test for the hypothesis $H_{0}:\pi =0$. The Wald test is given by

Wπ=π^′Z′Zπ^σ^v2=x′Z(Z′Z)−1Z′xσ^v2,

where π^=(Z′Z)−1Z′x is the first-stage OLS estimator, and σ^v2=x′MZx/n, where $M_{Z}=I-P_{Z}$. Under the null, $W_{\pi }\overset{d}{⟶}\chi_{k_{z}}^{2}$. The F-test is given by $F=W_{\pi }/k_{z}$. Note that we refrain from a degrees of freedom correction in the variance estimate. Also, note that the analyses here and further below extend to a model with additional exogenous regressors, as we can replace y, x and Z everywhere by their residuals from regressions on those exogenous regressors.

Staiger and Stock (1997) introduce weak instrument asymptotics as a local to zero alternative, $\pi =c/\sqrt{n}$, which ensures that the concentration parameter does not increase with the sample size

$$CP=\frac{\pi^{\prime }Z^{\prime }Z\pi }{\sigma_{v}^{2}}\overset{p}{⟶}\frac{c^{\prime }Q_{ZZ}c}{\sigma_{v}^{2}}\text{,}$$

where $Q_{ZZ}=\mathrm{plim}\left(n^{-1}Z^{\prime }Z\right)$.

Assuming that conditions are fulfilled, such that

$$\left(\begin{array}{c} \frac{1}{\sqrt{n}}Z^{\prime }u\\ \frac{1}{\sqrt{n}}Z^{\prime }v \end{array}\right)\overset{d}{⟶}\left(\begin{array}{c} \psi_{Zu}\\ \psi_{Zv} \end{array}\right)∼N\left(0,\Sigma ⊗Q_{ZZ}\right)\text{,}$$

and $k_{z}\geq 3$ when assessing relative bias. Then under weak instrument asymptotics,

β^2SLS−β=x′Z(Z′Z)−1Z′ux′Z(Z′Z)−1Z′x⟶dσuσv(λ+zv)′zu(λ+zv)′(λ+zv)

where

$$\lambda =\sigma_{v}^{-1}Q_{ZZ}^{1/2}c\text{;}\;z_{v}=\sigma_{v}^{-1}Q_{ZZ}^{-1/2}\psi_{Zv}\text{;}\;z_{u}=\sigma_{u}^{-1}Q_{ZZ}^{-1/2}\psi_{Zu}\text{.}$$

The bias of the OLS estimator is given by

β^OLS−β=x′ux′x=(Zπ+v)′u(Zπ+v)′(Zπ+v)=n−1(n−1/2c′Z′u+v′u)n−1(n−1c′Z′Zc+2n−1/2c′Z′v+v′v)⟶pplimn−1v′uplimn−1v′v=σuvσv2=σuσvρ,

where $\rho =\frac{\sigma_{uv}}{\sigma_{u}\sigma_{v}}$.

As a measure of relative bias, Stock and Yogo (2005) propose

Bn2=(E[β^2SLS]−βE[β^OLS]−β)2.

From the derivations above, and as $E\left[z_{u}|z_{v}\right]=\rho z_{v}$, it follows that

$$B_{n}^{2}={\left(E\left[\frac{{\left(\lambda +z_{v}\right)}^{\prime }z_{v}}{{\left(\lambda +z_{v}\right)}^{\prime }\left(\lambda +z_{v}\right)}\right]\right)}^{2}\text{,}$$

or

$$B_{n}=\left|E\left[\frac{{\left(\lambda +z_{v}\right)}^{\prime }z_{v}}{{\left(\lambda +z_{v}\right)}^{\prime }\left(\lambda +z_{v}\right)}\right]\right|\text{.}$$

Using weak instrument asymptotics, Stock and Yogo (2005) are therefore able to assess the size of the relative bias in relation to the first-stage F-statistic. As $z_{v}∼N\left(0,I_{k_{z}}\right)$, $B_{n}$ is determined by the values of λ and $k_{z}$. Let

$$l=\lambda^{\prime }\lambda /k_{z}=\frac{1}{k_{z}}\frac{c^{\prime }Q_{ZZ}c}{\sigma_{v}^{2}}\text{,}$$

then using Monte Carlo simulation, i.e. draws of $z_{v}∼N\left(0,I_{k_{z}}\right)$, Stock and Yogo (2005) find the values of lsuch that $B_{n}$ is a certain value, say 0.1, for different values of $k_{z}$. For example, when $k_{z}=4$and using 100,000 Monte Carlo draws, we obtain a relative expected bias $E\left[\frac{{\left(\lambda +z_{v}\right)}^{\prime }z_{v}}{{\left(\lambda +z_{v}\right)}^{\prime }\left(\lambda +z_{v}\right)}\right]=0.1$ for l=4.98. When $k_{z}=8$, we find l=7, again for $B_{n}=0.1$.

Using weak instrument asymptotics, Staiger and Stock (1997) derive the asymptotic distribution for the first-stage F-statistic, which is given by

$$F\overset{d}{⟶}\chi_{k_{z}}^{2}\left(k_{z}l\right)/k_{z}\text{,}$$

where $\chi_{k_{z}}^{2}\left(a\right)$ is the non-central chi-squared distribution with non-centrality parameter a. The F-test statistic can therefore be used to test the hypothesis

$$H_{0}:CP/k_{z}\leq l_{b}\;\text{vs}\;H_{1}:CP/k_{z}>l_{b}\text{,}$$

where $l_{b}$ is the value for l determined above such that the $B_{n}=b$. For b=0.1, we find from the scaled non-central chi-squared distribution a critical values of 10.20 when $k_{z}=4$ and 11.38 when $k_{z}=8$. In comparison, Stock and Yogo (2005), henceforth SY, find very similar critical values of 10.27 and 11.39 for these two cases respectively.

As an illustration, we performed a small simulation. The model is as in (1), (2), with β=1;

$$\left(\begin{array}{c} u_{i}\\ v_{i} \end{array}\right)∼N\left(\left(\begin{array}{c} 0\\ 0 \end{array}\right),\left(\begin{array}{cc} 1 & 0.5\\ 0.5 & 1 \end{array}\right)\right)\text{;}$$

the instruments in Z are four independent standard normally distributed random variables and $\pi ={\left(\begin{array}{cccc} c & c & c & c \end{array}\right)}^{\prime }/\sqrt{n}$, with c chosen such that the relative bias $B_{n}$ for n→∞ is equal to 0.1, or 10%. We set the sample size n=10,000 and show the results in Table 1 for 10,000 Monte Carlo replications. The results are clearly in line with the theory. The observed relative bias is just over 10% and the rejection frequency of the F-test using the SY weak instrument critical value is 5% at the 5% nominal level.

**Table 1:** Estimation and relative bias results for one-variable model.

	Mean	St dev	Rel bias	SY rej freq
β^OLS	1.4989	0.0086		
β^2SLS	1.0529	0.2173	0.1060	
F	5.97	2.36		0.0502

Notes: Sample size 10,000; 10,000 MC replications; β=1; F is the first-stage F-statistic for x; rel bias is the relative bias of the 2SLS estimator, relative to that of the OLS estimator; SY rej freq uses the 5% Stock–Yogo critical value for the F-test for a 10% relative bias.

The Wald test for testing the restriction $H_{0}:\beta =\beta_{0}$ is given by

W=(β^2SLS−β0)2(x′PZx)σ^u2,

where σ^u2=(y−xβ^2SLS)′(y−xβ^2SLS)/n. Staiger and Stock (1997) show that, under weak instrument asymptotics,

$$W\overset{d}{⟶}\frac{\nu_{2}^{2}/\nu_{1}}{1-2\rho \nu_{2}/\nu_{1}+{\left(\nu_{2}/\nu_{1}\right)}^{2}}\text{,}$$

where

$$\nu_{1}={\left(\lambda +z_{v}\right)}^{\prime }\left(\lambda +z_{v}\right)\text{;}\;\nu_{2}={\left(\lambda +z_{v}\right)}^{\prime }z_{u}\text{.}$$

The Wald size distortion is maximised for ρ=1, and SY find the critical values for the F-test such that the maximal size of the Wald test is a certain value, say 10%, at a nominal 5% level. For the Monte Carlo example above, we set ρ=1 and choose c such that the maximal size distortion of the Wald test is 10%, in which case the value of l is given by 16.415. The SY critical value in this case is given by 24.58. The results are given in Table 2, and confirm that the size of the Wald test is 10% and the rejection frequency of the F-test using the SY critical values is indeed 5%.

**Table 2:** Estimation and Wald test results for one-variable model.

	Mean	St dev	Rej freq	SY rej freq
β^OLS	1.9935	0.0008		
β^2SLS	1.0318	0.1184		
W	1.42	2.52	0.0994	
F	17.45	4.11		0.0501

Notes: Sample size 10,000; 10,000 MC replications; β=1,ρ=1; W is the Wald test for testing $H_{0}:\beta =1$; rej freq uses 5% critical value of $\chi_{1}^{2}$; SY rej freq uses the 5% Stock–Yogo critical value for the F-test, for a maximal 10% size of W.

## 3 Two variable model

Following the exposition in Angrist and Pischke (2009), we first consider the following two-variable model

(3) $$y=x_{1}\beta_{1}+x_{2}\beta_{2}+u$$

$$x_{1}=Z\pi_{1}+v_{1}$$

$$x_{2}=Z\pi_{2}+v_{2}$$

where y, $x_{1}$, $x_{2}$, u, $v_{1}$ and $v_{2}$ are n×1 vectors, with n the number of observations. Z is an $n\times k_{z}$ matrix of instruments, with $k_{z}\geq 2$ ($k_{z}\geq 4$ when assessing relative bias), and $\pi_{1}$ and $\pi_{2}$ are $k_{z}\times 1$ vectors. For an individual observation i,

$$\left(\begin{array}{c} u_{i}\\ v_{1i}\\ v_{2i} \end{array}\right)∼\left(0,\left(\begin{array}{cc} \sigma_{u}^{2} & \sigma_{Vu}^{\prime }\\ \sigma_{Vu} & \Sigma_{V} \end{array}\right)\right)\text{;}\;\Sigma_{V}=\left(\begin{array}{cc} \sigma_{1}^{2} & \sigma_{12}\\ \sigma_{12} & \sigma_{2}^{2} \end{array}\right)\text{.}$$

Equivalently, we can write

y=Xβ+uX=ZΠ+V

where $\beta ={\left(\beta_{1},\beta_{2}\right)}^{\prime }$; $X=\left(\begin{array}{cc} x_{1} & x_{2} \end{array}\right)$; $\Pi =\left(\begin{array}{cc} \pi_{1} & \pi_{2} \end{array}\right)$  and $V=\left(\begin{array}{cc} v_{1} & v_{2} \end{array}\right)$. Further, let x=vec(X), π=vec(Π) and v=vec(V).

The OLS estimates for $\pi_{j}$ are denoted π^j=(Z′Z)−1Z′xj, j=1,2, and the estimated variances are given by

Σ^V=(σ^12σ^12σ^12σ^22)=1nV^′V^=1n(v^1′v^1v^1′v^2v^1′v^2v^2′v^2),

where V^=X−ZΠ^.

The first-stage F-statistics are given by

Fj=π^j′Z′Zπ^jkzσ^j2=xj′Z(Z′Z)−1Z′xjkzσ^j2;j=1,2,

and $k_{z}F_{j}$ converges in distribution to a $\chi_{k_{z}}^{2}$ distribution under the null $H_{0}:\pi_{j}=0$. Significant first-stage F-statistics are clearly necessary, but not sufficient, for identification of β. For example, if $\pi_{1}=\delta \pi_{2}\neq 0$, both first-stage F-statistics will reject their null in large samples, but the model is clearly underidentified.

Staiger and Stock (1997) and Stock and Yogo (2005) consider weak instrument asymptotics where all reduced form parameters are local to zero, i.e.  $\Pi =C/\sqrt{n}$. The Wald test for $H_{0}:\pi =0$ is given by

Wπ=π^′(Σ^V−1⊗Z′Z)π^,

which is identical to the trace of the Cragg and Donald (1993) statistic

CD=Σ^V−1/2Π^′Z′ZΠ^Σ^V−1/2.

However, this Wald test statistic on the reduced form cannot be used in an equivalent way to assess relative bias and 2SLS Wald test size distortions as in the one-variable model above, because these are determined largely by the minimum eigenvalue of CD, $\tau_{\min}$. In other words, relative bias and Wald size distortions can be large if tr(CD) is large but $\tau_{\min}$ is small. In a general setting with g endogenous explanatory variables, $W_{\pi }=\mathrm{tr}\left(CD\right)$ is a test for $H_{0}:\mathrm{rank}\left(\Pi \right)=0$, whereas $\tau_{\min}$ is a test for $H_{0}:\mathrm{rank}\left(\Pi \right)=g-1$. SY derive critical values for $\tau_{\min}/k_{z}$ under the local to zero weak instrument asymptotics for maximal total relative bias and Wald test distortions, where the total relative bias is given by

(4) B2=(E[β^2SLS]−β)′ΣX(E[β^2SLS]−β)(E[β^OLS]−β)′ΣX(E[β^OLS]−β),

with $\Sigma_{X}=\mathrm{plim}\left(n^{-1}X^{\prime }X\right)$. In this case, as $\tau_{\min}$ is not the test statistic for $H_{0}:\pi =0$, unlike in the case of one endogenous variable, the correspondence is not exact and use of the SY critical values results in a conservative test in the sense that the null of weak instruments is rejected too infrequently when the null is true. This is not altogether an undesirable feature of the test, as a researcher can be quite confident that instruments are not weak when $\tau_{\min}/k_{z}$ is larger than the SY critical value.

The Staiger and Stock (1997) and Stock and Yogo (2005) results for the F-test and Cragg–Donald statistic in the one-variable and multiple-variable model respectively in relation to the relative bias and Wald test size distortions hold under the stated assumptions of the model and the reduced form equations for the endogenous variables. When the variances in the reduced forms are conditionally heteroskedastic, then one can compute robust F-statistics and the Kleibergen and Paap (2006) robust version of the Cragg–Donald statistic. These test statistics are then valid tests for underidentification as they have correct size under the null that the instruments are not informative, i.e. for testing that rank(Π)=0. But the documented relationship of the weak-instrument critical values and the sizes of the relative bias and Wald-test size distortion no longer holds, see for example Bun and de Haan (2010). This limits the exact use of the weak-instrument tests, as for example binary endogenous explanatory variables automatically produce a conditionally heteroskedastic reduced form. Also, this relationship brakes down in simple panel data models, when there is serial correlation in the reduced form errors, or indeed in simple time-series models with serial correlation. In our development of the conditional F-statistics for models with multiple endogenous variables, we maintain the same assumptions as Staiger and Stock (1997)  and Stock and Yogo (2005), and hence the same limitations. Olea and Pflueger (2013) have recently proposed an alternative robust F-test type procedure for weak instruments, but thus far it can only be applied to the one-endogenous variable model.

### 3.1 Conditional F-test

Angrist and Pischke (2009) propose an alternative conditional first-stage F-statistic for the case of multiple endogenous variables by reformulating the estimation problem to a one-variable model after replacing the other endogenous variables with their reduced form predictions. For instance, for the two-variable model, the 2SLS estimator for $\beta_{1}$ is obtained by 2SLS in the model

(5) y=x1β1+x^2β2+u∗,

where x^2=Zπ^2=PZx2, using Z as the instruments, and hence

β^1=(x1′Mx^2PZMx^2x1)−1x1′Mx^2PZy.

Therefore, β^1 can be seen as the 2SLS estimator in the one-variable model

(6) y=Mx^2x1β1+ξ,

where the residual Mx^2x1=x1−x^2δ˜, with δ˜=(x^2′x^2)−1x^2′x1, is instrumented by Z. The reduced form is then

(7) Mx^2x1=Zκ+ε

and the Angrist–Pischke F-statistic is testing the hypothesis $H_{0}:\kappa =0$, given by

(8) FAP=κ^′Z′Zκ^(kz−1)σ^ε2=x1′Mx^2PZMx^2x1(kz−1)σ^ε2,

where κ^ is the OLS estimator of κ,

κ^=(Z′Z)−1Z′Mx^2x1=(Z′Z)−1Z′(x1−x^2δ˜)=π^1−π^2δ˜;

and σ^ε2=ε^′ε^/n, with ε^=Mx^2x1−Zκ^. The degrees of freedom correction follows because x^2 has been predicted using the same instruments Z. If we partition $Z=\left[\begin{array}{cc} z_{1} & Z_{2} \end{array}\right]$  with $Z_{2}$ a $\left(k_{z}-1\right)\times n$ matrix, then the instrument set for (5) could equivalently be written as [x^2Z2].

As the problem seems to have been reduced to a one-endogenous variable model, $F_{AP}$ has been proposed to determine instrument strength for identification of individual structural parameters, like $\beta_{1}$ in the above derivation, and Stock and Yogo (2005) weak instrument critical values used to determine maximum relative bias of the IV estimator, relative to the OLS estimator for the single parameter. There are some issues with this, however, that seem to invalidate such an approach. Under the null that κ=0, $\left(k_{z}-1\right)F_{AP}$ does not follow an asymptotic $\chi_{k_{z}-1}^{2}$ distribution, unless $\pi_{1}=0$. An alternative F-statistic is easily derived that corrects for this, but the relative bias results as described in the previous section for the one-variable model do not carry over to the individual parameters in this multiple endogenous variables model.

To consider the asymptotic distribution, for any given value of δ we have that

x1−x^2δ=x1−x2δ+(x2−x^2)δ=Z(π1−δπ2)+v1−δv2+δMZv2=Z(π1−δπ2)+v1−δPZv2.

Clearly, the OLS estimator for $\kappa_{\delta }$ in the model

(9) x1−x^2δ=Zκδ+ε∗

is given by

κ^δ=(Z′Z)−1Z′(x1−x^2δ)=(Z′Z)−1Z′(x1−x2δ)=π^1−δπ^2=π1−δπ2+(Z′Z)−1Z′(v1−δv2)

and hence the variance of the OLS estimator is given by

(10) Var(κ^δ)=(σ12−2δσ12+δ2σ22)(Z′Z)−1.

The F-statistic for testing $H_{0}:\kappa_{\delta }=0$ in (9) is

Fδ=κ^′Z′Zκ^kz(σ^12−2δσ^12+δ2σ^22),

and $k_{z}F_{\delta }$ converges in distribution to a $\chi_{k_{z}}^{2}$ distribution under the null that $\kappa_{\delta }=0$, or $\pi_{1}=\delta \pi_{2}$. However, computing the standard F-test statistic in (9) as

Fs=κ^′Z′Zκ^kzσ^ε∗2

does not result in $F_{\delta }$ as

ε^∗′ε^∗=(x1−x^2δ)′MZ(x1−x^2δ)=x1′MZx1=v^1′v^1

and hence

Fs=κ^′Z′Zκ^kzσ^12.

Therefore the denominator of $F_{s}$ does not estimate the variance as in (10) correctly and $k_{z}F_{s}$ does not converge to a $\chi_{k_{z}}^{2}$ distribution under the null, unless δ=0. The correct F-statistic would be obtained by the standard F-test if the dependent variable in (9) was $x_{1}-\delta x_{2}$ instead of x1−δx^2.

The Angrist–Pischke approach does replace δ by an estimate $\overset{˜}{\delta }$. By developing a formal testing framework we show that the same issues arise and that $\left(k_{z}-1\right)F_{AP}$ does not have an asymptotic $\chi_{k_{z}-1}^{2}$ distribution under the null that $\pi_{1}=\delta \pi_{2}$, unless δ=0.

Partition $Z=\left[\begin{array}{cc} z_{1} & Z_{2} \end{array}\right]$. We can write the reduced from for $x_{1}$ as

(11) $$x_{1}=Z\pi_{1}+v_{1}=Z\pi_{2}+Z\left(\pi_{1}-\pi_{2}\right)+v_{1}=Z\pi_{2}\delta +Z_{2}\left(\pi_{12}-\pi_{22}\delta \right)+v_{1}=x_{2}\delta +Z_{2}\left(\pi_{12}-\pi_{22}\delta \right)+v_{1}-\delta v_{2}$$

where $\pi_{1}$ and $\pi_{2}$ are partitioned as ${\left[\begin{array}{cc} \pi_{11} & \pi_{12}^{\prime } \end{array}\right]}^{\prime }$and ${\left[\begin{array}{cc} \pi_{21} & \pi_{22}^{\prime } \end{array}\right]}^{\prime }$respectively; $\delta =\pi_{11}/\pi_{21}$, implicitly assuming that $\pi_{21}\neq 0$. Hence a test for underidentification is a test for $H_{0}:\gamma =0$, in the model

(12) $$x_{1}=x_{2}\delta +Z_{2}\gamma +v^{*}\text{,}$$

where $v^{*}=v_{1}-\delta v_{2}$. Clearly, $x_{2}$ is an endogenous variable in (12), but we can estimate the parameters δ and γ by IV, using Z as instruments. The 2SLS estimators for δ and γ are given by

δ^=(x^2′MZ2x^2)−1x^2′MZ2x1;γ^=(Z2′Mx^2Z2)−1Z2′Mx^2x1

and

Var(γ^)=σv∗2(Z2′Mx^2Z2)−1,

with $\sigma_{v^{*}}^{2}=\sigma_{1}^{2}-2\delta \sigma_{12}+\delta^{2}\sigma_{2}^{2}$. The F-test statistic for testing $H_{0}:\gamma =0$ is therefore given by

Fγ=x1′Mx^2Z2(Z2′Mx^2Z2)−1Z2′Mx^2x1(kz−1)(v^∗′v^∗/n)

with

v^∗=x1−x2δ^−Z2γ^=Zπ^1+v^1−Zπ^2δ^−δ^v^2−Z2γ^=v^1−δ^v^2,

as the IV estimates are given by

δ^=π^11π^21;γ^=π^12−π^22δ^.

Hence,

σ^v∗2=1nv^∗′v^∗=σ^12−2δ^σ^12+δ^2σ^22

is a consistent estimator of $\sigma_{v^{*}}^{2}$.

The Angrist and Pischke (2009)F-statistic as described above is related to $F_{\gamma }$, as

FAP=x1′Mx^2Z(Z′Z)−1Z′Mx^2x1(kz−1)(ε^′ε^/n)=x1′Mx^2Z2(Z2′Mx^2Z2)−1Z2′Mx^2x1(kz−1)σ^12,

because

x1′Mx^2PZMx^2x1=x1′PZMx^2PZx1=x^1′Mx^2x^1=γ^Z2′Mx^2Z2γ^=x1′Mx^2Z2(Z2′Mx^2Z2)−1Z2′Mx^2x1,

and the sum of squared residuals is given by

ε^′ε^=x1′Mx^2MZMx^2x1=x1′MZx1=v^1′v^1

and hence ε^′ε^/n=σ^12. Therefore, whilst the numerators are the same in $F_{AP}$ and $F_{\gamma }$, the denominators are different. $\left(k_{z}-1\right)F_{AP}$ is therefore not asymptotically $\chi_{k_{z}-1}^{2}$ distributed under the null, $H_{0}:\pi_{1}=\delta \pi_{2}$, unless δ=0 and hence $\pi_{1}=0$.

Clearly, δ˜=(x^2′x^2)−1x^2′x1 is an estimate of δ under the null that $\pi_{1}=\delta \pi_{2}$ and hence γ=0. Let ${\overset{˜}{v}}^{*}=x_{1}-x_{2}\overset{˜}{\delta }$ be the residual under the null, then the LM test for the null $H_{0}:\gamma =0$ is given by

$$LM=\frac{{\overset{˜}{v}}^{*\prime }Z{\left(Z^{\prime }Z\right)}^{-1}Z^{\prime }{\overset{˜}{v}}^{*}}{{\overset{˜}{v}}^{*\prime }{\overset{˜}{v}}^{*}/n}$$

which converges to a $\chi_{k_{z}-1}^{2}$ distribution under the null. LM is equal to $nR^{2}$ in the model

(13) $$x_{1}-x_{2}\overset{˜}{\delta }=Z\kappa +\xi \text{.}$$

The F-test in (13), with appropriate degrees of freedom correction, is given by

(14) F1|2=κ^′Z′Zκ^(kz−1)(ξ^′ξ^/n)=(π^1−δ˜π^2)′Z′Z(π^1−δ˜π^2)(kz−1)(σ^12+δ˜2σ^22−2δ˜σ^12)=x1′Mx^2Z2(Z2′Mx^2Z2)−1Z2′Mx^2x1(kz−1)(σ^12+δ˜2σ^22−2δ˜σ^12),

which is only different from $F_{\gamma }$ through the estimate of δ in the denominator. In $F_{1|2}$ this is invariant to which instrument has been excluded from Z in forming $Z_{2}$, making it therefore preferable to $F_{\gamma }$. Clearly, $F_{1|2}$ differs from $F_{AP}$ by using the IV residual $x_{1}-x_{2}\overset{˜}{\delta }$ in (13) instead of the second stage residual x1−x^2δ˜ for $F_{AP}$ in (7).

Analogous to (11), we can write for $x_{2}$

$$x_{2}=x_{1}\delta^{*}+Z_{2}\left(\pi_{22}-\pi_{21}\delta^{*}\right)+v_{2}-\delta^{*}v_{1}=x_{1}\delta^{*}+Z_{2}\gamma^{*}+v^{**}$$

where $\delta^{*}=\pi_{12}/\pi_{22}=\delta^{-1}$, $\gamma^{*}=-\gamma /\delta$ and $v^{**}=v^{*}/\delta$. Clearly

F2|1=(π^2−δ˜∗π^1)′Z′Z(π^2−δ˜∗π^1)(kz−1)(σ^22+δ˜∗2σ^12−2δ˜∗σ^12),

where δ˜∗=(x^1′x^1)−1x^1′x2, has the same asymptotic properties as $F_{1|2}$ under $H_{0}:\pi_{1}=\delta \pi_{2}$, but it is not identical to $F_{1|2}$ as ${\overset{˜}{\delta }}^{*}\neq {\overset{˜}{\delta }}^{-1}$.

### 3.2 Relationship with Cragg–Donald statistic

With g endogenous variables, the minimum eigenvalue of the Cragg–Donald statistic, $\tau_{\min}$, is a test for $H_{0}:\mathrm{rank}\left(\Pi \right)=g-1$ against the alternative $H_{1}:\mathrm{rank}\left(\Pi \right)=g$. For the two-variable model, this null is of course equivalent to $H_{0}:\pi_{1}=\delta \pi_{2}$. The Cragg–Donald test is based on the restricted estimates under the null, using the minimum-distance criterion,

$$\left(\bar{\delta },{\bar{\pi }}_{2}\right)=\underset{\delta ,\pi_{2}}{\arg\min}H\left(\delta ,\pi_{2}\right)\text{,}$$

with

H(δ,π2)=((π^1π^2)−(δπ2π2))′(Σ^−1⊗Z′Z)((π^1π^2)−(δπ2π2)).

The Cragg–Donald test statistic is then

$$\tau_{\min}=H\left(\bar{\delta },{\bar{\pi }}_{2}\right)\overset{d}{⟶}\chi_{k_{z}-1}^{2}$$

under the null. We show in the Appendix that

H(δ¯,π¯2)=(π^1−δ¯π^2)′Z′Z(π^1−δ¯π^2)σ^12+δ¯2σ^22−2δ¯σ^12

and hence the only difference between $F_{1|2}$, $F_{2|1}$ and $\tau_{\min}/\left(k_{z}-1\right)$ is the estimate for δ. Clearly, unlike the F-statistics, $\tau_{\min}$ is invariant to normalisation, as $H\left({\bar{\delta }}^{*},{\bar{\pi }}_{1}\right)=H\left(\bar{\delta },{\bar{\pi }}_{2}\right)$. Because of this, computation of both $F_{1|2}$ and $F_{2|1}$ can provide further information about the nature of the weakness of the instruments, as their values can indicate whether the rank reduction is due to e.g.  $\pi_{1}=0$ (δ=0), which $\tau_{\min}$ cannot distinguish, as we will show below. We will also present a three-endogenous variables example in Section  4.3 which further highlights the additional information about instrument strength revealed by the three conditional F-statistics relative to that of the Cragg–Donald statistic.

## 4 Local to rank one weak instrument asymptotics in the two-variable model

In the previous section, we have shown that $\left(k_{z}-1\right)F_{\gamma }$ has a limiting $\chi_{k_{z-1}}^{2}$ distribution under the null that γ=0 in (12). We next investigate whether $F_{\gamma }$ can be used to assess whether instruments are weak for individual parameters as described in Section  2. We focus in the derivation below on $F_{\gamma }$ as the setup for this test is easier to use with our weak instruments asymptotics, but results of course carry over directly to $F_{1|2}$, $F_{2|1}$ and $\tau_{\min}$.

We are interested in the case that the instruments are not weak for each equation, but where the rank of Π approaches a rank reduction of one. We specify LRR1 weak instrument asymptotics as $\gamma =c/\sqrt{n}$, or

$$\pi_{12}=\delta \pi_{22}+c/\sqrt{n}\text{.}$$

We can then write the reduced form of $x_{1}$ as

x1=Zπ2δ+Z2(π12−δπ22)+v1=x^2δ+Z2c/n+(v1−δPZv2).

The IV estimator for $\beta_{1}$ is given by

β^1,2SLS=x1′Mx^2Z2(Z2′Mx^2Z2)−1Z2′Mx^2yx1′Mx^2Z2(Z2′Mx^2Z2)−1Z2′Mx^2x1

and it follows that

β^1,2SLS−β1=x1′Mx^2Z2(Z2′Mx^2Z2)−1Z2′Mx^2ux1′Mx^2Z2(Z2′Mx^2Z2)−1Z2′Mx^2x1

as Mx^2x^2=0, Mx^2MZ=(I−PZx2(x2′PZx2)−1x2′PZ)MZ=MZ, and hence Z2′Mx^2MZv2=Z2′MZv2=0.

We assume that

(1nZ2′Mx^2u1nZ2′Mx^2(v1−δv2))⟶d(ψZ2∗uψZ2∗(v1−δv2))=N(0,Ω⊗Q),

where

Ω=(σu2σu1−δσu2σu1−δσu2σ12+δ2σ22−2δσ12);Z2∗=Mx^2Z2;Q=plim(n−1Z2∗′Z2∗).

It is then easily shown that

x1′Mx^2Z2(Z2′Mx^2Z2)−1Z2′Mx^2x1⟶dσv1−δv22(λ˜+z˜v)′(λ˜+z˜v)

and

x1′Mx^2Z2(Z2′Mx^2Z2)−1Z2′Mx^2u⟶dσuσv1−δv2(λ˜+z˜v)′z˜u

where

$$\sigma_{v_{1}-\delta v_{2}}=\sqrt{\sigma_{1}^{2}+\delta^{2}\sigma_{2}^{2}-2\delta \sigma_{12}};\overset{˜}{\lambda }=\sigma_{v_{1}-\delta v_{2}}^{-1}Q^{1/2}c\text{;}\;{\overset{˜}{z}}_{v}=\sigma_{v_{1}-\delta v_{2}}^{-1}Q^{-1/2}\psi_{Z_{2}^{*}\left(v_{1}-\delta v_{2}\right)}\text{;}\;{\overset{˜}{z}}_{u}=\sigma_{u}^{-1}Q^{-1/2}\psi_{Z_{2}^{*}u}\text{.}$$

We are therefore in the same setup as Staiger and Stock (1997) and Stock and Yogo (2005), and the distribution of the bias of β^1,2SLS is given by

β^1,2SLS−β1=x1′Mx^2Z2(Z2′Mx^2Z2)−1Z2′Mx^2ux1′Mx^2Z2(Z2′Mx^2Z2)−1Z2′Mx^2x1⟶dσuσv1−δv2(λ˜+z˜v)′z˜u(λ˜+z˜v)′(λ˜+z˜v),

and

E(β^1,2SLS)−β1⟶σu1−δσu2σ12+δ2σ22−2δσ12E((λ˜+z˜v)′z˜v(λ˜+z˜v)′(λ˜+z˜v)).

One would therefore think that one could proceed as in the one-variable model as specified above, with

$$\overset{˜}{l}=\overset{˜}{\lambda }{}^{\prime }\overset{˜}{\lambda }/\left(k_{z}-1\right)=\frac{1}{k_{z}-1}\frac{c^{\prime }Qc}{\sigma_{1}^{2}+\delta^{2}\sigma_{2}^{2}-2\delta \sigma_{12}}$$

and the critical values from the non-central chi-squared distribution applied to

Fγ=x1′Mx^2Z2(Z2′Mx^2Z2)−1Z2′Mx^2x1(kz−1)(σ^12+δ^2σ^22−2δ^σ^12).

However, in this case the bias of the OLS estimator of $\beta_{1}$ in the model

$$y=x_{1}\beta_{1}+x_{2}\beta_{2}+u$$

is given by

β^1,OLS−β1=x1′Mx2ux1′Mx2x1.

As

$$x_{1}=x_{2}\delta +Z_{2}c/\sqrt{n}+\left(v_{1}-\delta v_{2}\right)\text{,}$$

we get that

$$\mathrm{plim}\left(n^{-1}\left(x_{1}^{\prime }M_{x_{2}}u\right)\right)=\mathrm{plim}\left(n^{-1}\left(\frac{c}{\sqrt{n}}Z_{2}^{\prime }M_{x_{2}}u+{\left(v_{1}-\delta v_{2}\right)}^{\prime }M_{x_{2}}u\right)\right)\text{.}$$

Further,

$$\mathrm{plim}\left(n^{-1}\left(x_{1}^{\prime }M_{x_{2}}x_{1}\right)\right)=\mathrm{plim}\left(n^{-1}\left(\frac{c}{\sqrt{n}}Z_{2}^{\prime }M_{x_{2}}Z_{2}\frac{c}{\sqrt{n}}+2\frac{c^{\prime }}{\sqrt{n}}Z_{2}^{\prime }M_{x_{2}}\left(v_{1}-\delta v_{2}\right)+{\left(v_{1}-\delta v_{2}\right)}^{\prime }M_{x_{2}}\left(v_{1}-\delta v_{2}\right)\right)\right)\text{.}$$

From these results we find that the bias of the OLS estimator converges to

plim(β^1,OLS−β1)=plim(n−1(v1−δv2)′Mx2u)plim(n−1(v1−δv2)′Mx2(v1−δv2))=σu1−δσu2−(σ12−δσ22)σu2π2′QZZπ2+σ22σ12+δ2σ22−2δσ12−(σ12−δσ22)2π2′QZZπ2+σ22

and therefore, we now have that

Bn,1=|E[β^1,2SLS]−β1||E[β^1,OLS]−β1|≠|E[(λ˜+z˜v)′z˜v(λ˜+z˜v)′(λ˜+z˜v)]|

and so the direct relationship between the relative bias of the individual parameter and the value of the concentration parameter does not hold in this setting.^1^

However, we can get a result for the total relative bias. First of all, we show in the Appendix that for the 2SLS estimator for $\beta_{2}$ the following holds,

β^2,2SLS−β2⟶d−δσuσv1−δv2(λ˜+z˜v)′z˜u(λ˜+z˜v)′(λ˜+z˜v),

and hence, asymptotically,

E[β^2,2SLS]−β2=−δ(E[β^1,2SLS]−β1).

From this it follows that β^2,2SLS is consistent when δ=0, that is in the situation where the instruments are strong for $x_{2}$, but weak for $x_{1}$ in the sense that $\pi_{1}$ is local to zero.

We then show in the Appendix that

B2=(E[β^2SLS]−β)′ΣX(E[β^2SLS]−β)(E[β^OLS]−β)′ΣX(E[β^OLS]−β)≤b2,

where

$$b=E\left(\frac{{\left(\overset{˜}{\lambda }+{\overset{˜}{z}}_{v}\right)}^{\prime }{\overset{˜}{z}}_{v}}{{\left(\overset{˜}{\lambda }+{\overset{˜}{z}}_{v}\right)}^{\prime }\left(\overset{˜}{\lambda }+{\overset{˜}{z}}_{v}\right)}\right)\text{.}$$

From this it follows that we can use the SY critical values for $\tau_{\min}/\left(k_{z}-1\right)$, $F_{1|2}$ and $F_{2|1}$ to assess LRR1 weak instrument maximal total relative bias. These are the critical values tabulated for the one-endogenous variable case with $k_{z}-1$ instruments.

We can also use the equivalent SY critical values for assessing the maximal size of the individual 2SLS Wald tests. We get for the Wald test for the simple null $H_{0}:\beta_{1}=\beta_{1}^{0}$

W1=(β^1,2SLS−β10)2(x1′Mx^2Z2(Z2′Mx^2Z2)−1Z2′Mx^2x1)σ^u2=σu2σ^u2((λ˜+z˜v)′z˜u)2(λ˜+z˜v)′(λ˜+z˜v)

where

σ^u2=(y−x1β^1,2SLS−x2β^2,2SLS)′(y−x1β^1,2SLS−x2β^2,2SLS)/n.

We find that

σ^u2⟶dσu2(1−2σu1−δσu2σuσv1−δv2ν˜2ν˜1+(ν˜2v˜1)2),

where

$${\overset{˜}{\nu }}_{1}={\left(\overset{˜}{\lambda }+{\overset{˜}{z}}_{v}\right)}^{\prime }\left(\overset{˜}{\lambda }+{\overset{˜}{z}}_{v}\right)\text{;}\;{\overset{˜}{\nu }}_{2}={\left(\overset{˜}{\lambda }+{\overset{˜}{z}}_{v}\right)}^{\prime }{\overset{˜}{z}}_{u}\text{.}$$

The Wald test is then, as in Staiger and Stock (1997) and Stock and Yogo (2005), equal to

$$W_{1}=\frac{{\overset{˜}{v}}_{2}^{2}/{\overset{˜}{v}}_{1}}{1-2\overset{˜}{\rho }{\overset{˜}{\nu }}_{2}/{\overset{˜}{\nu }}_{1}+{\left({\overset{˜}{\nu }}_{2}/{\overset{˜}{\nu }}_{1}\right)}^{2}}$$

where $\overset{˜}{\rho }=\frac{\sigma_{u1}-\delta \sigma_{u2}}{\sigma_{u}\sigma_{v_{1}-\delta v_{2}}}$, and so we can again use the SY critical values for the F-statistic for maximal size of the Wald-test, achieved when $\overset{˜}{\rho }=1$. Clearly, we get the same results for $W_{2}$, the Wald test for $H_{0}:\beta_{2}=\beta_{2}^{0}$.

### 4.1 Monte Carlo illustration

To illustrate, we generate data from the model as specified above, with

$$\left(\begin{array}{c} u_{i}\\ v_{1i}\\ v_{2i} \end{array}\right)∼N\left(\left(\begin{array}{c} 0\\ 0\\ 0 \end{array}\right),\left(\begin{array}{ccc} \sigma_{u}^{2} & \sigma_{u1} & \sigma_{u2}\\ \sigma_{u1} & \sigma_{1}^{2} & \sigma_{12}\\ \sigma_{u2} & \sigma_{12} & \sigma_{2}^{2} \end{array}\right)\right)\text{.}$$

The instruments are drawn independently from the standard normal distribution, with $k_{z}=4$, and hence $Q_{ZZ}=I_{4}$. We set $\pi_{2}={\left(-0.5,0.5,-0.5,0.5\right)}^{\prime }$ and $\pi_{1}=\delta \pi_{2}+{\left(0,c,c,c\right)}^{\prime }/\sqrt{n}$. We have

Q=plim1nZ2′Mx^2Z2=Ikz−1−π22π22′π2′π2,

where $\pi_{2}={\left[\begin{array}{cc} \pi_{21} & \pi_{22} \end{array}\right]}^{\prime }$ is partitioned commensurate with $Z=\left[\begin{array}{cc} z_{1} & Z_{2} \end{array}\right]$.

The limit of the concentration parameter for this specific configuration is given by

$$CPl=\frac{c^{\prime }Qc}{\sigma_{1}^{2}+\delta^{2}\sigma_{2}^{2}-2\delta \sigma_{12}}=\frac{3c^{2}-\frac{c^{2}}{4}}{\sigma_{1}^{2}+\delta^{2}\sigma_{2}^{2}-2\delta \sigma_{12}}\text{.}$$

We choose c such that the concentration parameter has the value for which the IV estimator for β has a maximal total relative bias of 10%. We have further set the parameters as follows: $\beta_{1}=0.5;\;\beta_{2}=-0.3;\;\sigma_{u}^{2}=\sigma_{1}^{2}=\sigma_{2}^{2}=1$; $\sigma_{u1}=0.1;\;\sigma_{u2}=-0.7;\;\sigma_{12}=-0.7$ and δ=0.7. This design is such that the additional terms in the OLS bias are important, with

$$\frac{\sigma_{u1}-\delta \sigma_{u2}}{\sigma_{1}^{2}+\delta^{2}\sigma_{2}^{2}-2\delta \sigma_{12}}\frac{\sigma_{1}^{2}+\delta^{2}\sigma_{2}^{2}-2\delta \sigma_{12}-\frac{{\left(\sigma_{12}-\delta \sigma_{2}^{2}\right)}^{2}}{\pi_{2}^{\prime }Q_{ZZ}\pi_{2}+\sigma_{2}^{2}}}{\sigma_{u1}-\delta \sigma_{u2}-\frac{\left(\sigma_{12}-\delta \sigma_{2}^{2}\right)\sigma_{u2}}{\pi_{2}^{\prime }Q_{ZZ}\pi_{2}+\sigma_{2}^{2}}}=3.5591$$

i.e. the OLS bias for $\beta_{1}$ is much smaller than $\frac{\sigma_{u1}-\delta \sigma_{u2}}{\sigma_{1}^{2}+\delta^{2}\sigma_{2}^{2}-2\delta \sigma_{12}}$. The results are given in Table 3 for a sample size of 10,000 observations. The individual standard F-statistics are very large. As expected, the IV estimator of $\beta_{1}$ has a large relative bias of 0.3441, approximately equal to 3.56∗0.1, but the relative bias of $\beta_{2}$ is much smaller at 0.0498. The distributions of $F_{1|2}$, $F_{2|1}$ and $\tau_{\min}/\left(k_{z}-1\right)$ are virtually identical, each with a mean of 4.7 and rejection frequency of 4.6% at the 5% nominal level using the weak instrument critical value. In comparison, the AP F-statistics are much larger in this case with the mean of $F_{AP,1}$ equal to 11.82, and that of $F_{AP,2}$ equal to 22.93.

**Table 3:** Estimation results and relative bias for two-variable model.

	Mean	St dev	Rel bias	SY rej freq
β^1,OLS	0.5695	0.0070		
β^2,OLS	−0.6506	0.0062		
β^1,2SLS	0.5239	0.1979	0.3441	
β^2,2SLS	−0.3174	0.1419	0.0498	
$F_{1}$	1290	44		
$F_{2}$	2503	71		
$F_{AP,1}$	11.82	5.91		0.6256
$F_{AP,2}$	22.93	11.46		0.9082
$F_{1|2}$	4.70	2.35		0.0460
$F_{2|1}$	4.71	2.36		0.0464
$\tau_{\min}/\left(k_{z}-1\right)$	4.70	2.35		0.0457
$\tau_{\min}/k_{z}$	3.52	1.76		0.0267

Notes: Sample size 10,000; 10,000 MC replications; $\beta_{1}=0.5$; $\beta_{2}=-0.3$; $F_{j}$ is the first-stage F-statistic for $x_{j},j=1,2$; $F_{AP,j}$ is the Angrist–Pischke F-statistic and $F_{1|2}$  and $F_{2|1}$  are the conditional F-statistics as in (14); $\tau_{\min}$ is the Cragg–Donald minimum eigenvalue statistic; rel bias is the relative bias of the 2SLS estimator, relative to that of the OLS estimator; SY rej freq uses the 5% Stock–Yogo critical values for a maximum 10% total relative bias.

The total relative bias in this design is found to be equal to 7.6%, which is less than 10%, as predicted by the theory above. The SY test for weak instruments for Π local to 0 is conservative and has a rejection frequency of 2.6%. This test is given by $\tau_{\min}/k_{z}$ and the weak instrument critical value is derived for two endogenous variables with $k_{z}$ instruments. In contrast, the weak instrument critical values for $F_{1|2}$, $F_{2|1}$ and $\tau_{\min}/\left(k_{z}-1\right)$ are those for one endogenous variable with $k_{z}-1$ instruments. From Table 1 in SY, it is easily established that when $\tau_{\min}/k_{z}$ is larger than its associated tabulated critical value, then $\tau_{\min}/\left(k_{z}-1\right)$ is also larger than its weak instrument critical value, so we would always reject LRR1 weak instrument problems whenever we reject rank zero weak instrument problems.

In Table 4 we present results for the Wald test statistics in a design with $\overset{˜}{\rho }=1$, by changing the variance parameters to $\sigma_{u1}=0.755$, $\sigma_{u2}=0.35$ and $\sigma_{12}=-0.35$, again choosing c such that the size of the Wald tests is 10% at the 5% level. The simulations confirm the analytical results. The rejection frequencies of the Wald tests are just over 10% and the rejection frequencies of $F_{1|2}$, $F_{2|1}$ and $\tau_{\min}/\left(k_{z}-1\right)$ just over 5%. In this case, the SY weak instrument test $\tau_{\min}/k_{z}$ using the tabulated critical value for two endogenous variables and four instruments is also just over 5%.

**Table 4:** Estimation and Wald tests results for two-variable model.

	Mean	St dev	Rej freq	SY rej freq
β^1,OLS	1.4990	0.0007		
β^2,OLS	0.3899	0.0006		
β^1,2SLS	0.5257	0.1565		
β^2,2SLS	−0.2827	0.1071		
$W_{1}$	1.47	2.86	0.1016	
$W_{2}$	1.46	2.87	0.1017	
$W_{12}$	2.61	3.58	0.1080	
$F_{1|2}$	14.85	4.40		0.0548
$F_{2|1}$	14.93	4.45		0.0585
$\tau_{\min}/\left(k_{z}-1\right)$	14.84	4.40		0.0517
$\tau_{\min}/k_{z}$	11.13	3.30		0.0545

Notes: Sample size 10,000; 10,000 MC replications; $\beta_{1}=0.5$; $\beta_{2}=-0.3\;W_{j}$  is the Wald test for $H_{0}{:\beta }_{j}{=\beta }_{0j}$; $W_{12}$ is joint Wald test; $F_{1|2}$ and $F_{2|1}$ are the conditional F-statistics as in (14); $\tau_{\min}$ is the Cragg–Donald minimum eigenvalue statistic; rej freq for Wald tests uses 5% critical value of $\chi^{2}$ distribution; SY rej freq uses the 5% Stock–Yogo critical values for a maximal 10% size of Wald tests.

### 4.2 The case δ=0

When δ=0, we have in the process above that $\pi_{1}$ is local to zero, and hence the instruments for $x_{1}$ are weak, but not for $x_{2}$. As shown above, β^2,2SLS is in this case consistent for $\beta_{2}$, but β^1,2SLS will suffer from a weak instrument bias. This situation may actually be of interest if the main research focus is on the effect of $x_{2}$ on y. If the instruments used are then strong for $x_{2}$ but weakly or not informative for $x_{1}$, the IV estimator for $\beta_{2}$ will be well behaved. In Table 5, we show the results for the bias of the 2SLS estimates, for when δ=0 and where we have further set $\sigma_{u1}=0.8$. All other parameters remain the same as for the results presented in Table 3, and we have set the value of c again such that the maximum total relative bias is 10%. As can be seen from the table, the results are as expected. The value of the first-stage F-statistic for $x_{1}$, $F_{1}$ is now small, whilst that of $F_{2}\;$  is large. The behaviour of $F_{AP,1}$ is now the same as that of $F_{1|2}$, both rejecting the null of weak instruments 5% of the time using the SY critical values for $k_{z}-1$ instruments. β^2,2SLS is consistent, but the total relative bias is at 9.7% only just below 10%.

**Table 5:** Estimation results and relative bias for two-variable model, δ=0.

	Mean	St dev	Rel bias	SY rej freq
β^1,OLS	1.2317	0.0067		
β^2,OLS	−0.3976	0.0047		
β^1,2SLS	0.5776	0.3001	0.0776	
β^2,2SLS	−0.3010	0.0103	−0.0010	
$F_{1}$	4.08	1.88		0.0044
$F_{2}$	2503	70		1.0000
$F_{AP,1}$	4.79	2.39		0.0515
$F_{AP,2}$	2922	502		1.0000
$F_{1|2}$	4.72	2.36		0.0474
$F_{2|1}$	462	1184		0.8811
$\tau_{\min}/\left(k_{z}-1\right)$	4.72	2.36		0.0470
$\tau_{\min}/k_{z}$	3.54	1.77		0.0259

Notes: Sample size 10,000; 10,000 MC replications; $\beta_{1}=0.5$; $\beta_{2}=-0.3\;F_{j}$  is the first-stage reduced form F-statistic for $x_{j},j=1,2$; $F_{AP,j}$  is the Angrist–Pischke F-statistic and $F_{1|2}$  and $F_{2|1}$  are the conditional F-statistics as in (14); $\tau_{\min}$ is the Cragg–Donald minimum eigenvalue statistic; rel bias is the relative bias of the 2SLS estimator, relative to that of the OLS estimator; SY rej freq uses the 5% Stock–Yogo critical values for a maximum 10% total relative bias.

### 4.3 More than two endogenous variables

As is clear from the analyses above for the two-variable model, the use of $F_{1|2}$ and $F_{2|1}$ under our LRR1 weak instrument asymptotics do not reveal more information than the Cragg–Donald statistic $\tau_{\min}/\left(k_{z}-1\right)$, unless δ=0 and hence $\pi_{1}$ is local to zero. The derivations for the two-variable model easily extend to the general case of several endogenous variables. The computation of the individual conditional F-statistics could then reveal further interesting patterns that the Cragg–Donald statistic will not be able to. For example, consider a three-variable model, which has a local rank reduction of one, of the form

$$\pi_{1}=\delta_{2}\pi_{2}+\delta_{3}\pi_{3}+c/\sqrt{n}$$

but with $\delta_{3}=0$. The conditional F-statistics are in this case computed from

$$x_{j}-X_{-j}\overset{˜}{\delta }=Z\kappa +\xi \text{,}$$

where $X_{-j}$ is the matrix of endogenous variables with $x_{j}\;$  excluded and δ˜=(X^−j′X^−j)−1X^−j′xj. The conditional F-statistics are then

(15) Fxj|X−j=κ^′Z′Zκ^(kz−2)(ξ^′ξ^/n),

see the Appendix for simple Stata code to calculate $F_{x_{j}|X_{-j}}$.

Table 6 presents some simulation results for this particular case for the following design

$$\left(\begin{array}{c} u_{i}\\ v_{1i}\\ v_{2i}\\ v_{3i} \end{array}\right)∼N\left(\left(\begin{array}{c} 0\\ 0\\ 0\\ 0 \end{array}\right),\left(\begin{array}{cccc} 1 & 0.8 & 0.3 & 0.6\\ 0.8 & 1 & 0.3 & 0.5\\ 0.3 & 0.3 & 1 & 0.4\\ 0.6 & 0.5 & 0.4 & 1 \end{array}\right)\right)\text{,}$$

$\delta_{2}=0.5$; $\delta_{3}=0$; $\beta_{1}=0.5$; $\beta_{2}=-0.3$; $\beta_{3}=0.7$. The instruments are again drawn independently form the standard normal distribution, with $k_{z}=5$, and c is again chosen such that the total relative bias is less than 10%.

**Table 6:** Estimation results and relative bias for three-variable model.

	Mean	St dev	Rel bias	SY rej freq
β^1,OLS	1.1337	0.0068		
β^2,OLS	−0.4581	0.0050		
β^3,OLS	0.9526	0.0055		
β^1,2SLS	0.5709	0.3086	0.1120	
β^2,2SLS	−0.3361	0.1575	0.2285	
β^3,2SLS	0.6990	0.0161	−0.0040	
$F_{1}$	650	26		
$F_{2}$	2504	67		
$F_{3}$	902	32		
$F_{1|2,3}$	4.82	2.38		0.0514
$F_{2|1,3}$	4.84	2.41		0.0531
$F_{3|1,2}$	198.21	329.06		0.8779
$\tau_{\min}/\left(k_{z}-2\right)$	4.82	2.38		0.0513
$\tau_{\min}/k_{z}$	2.89	1.43		0.0156

Notes: Sample size 10,000; 10,000 MC replications; $\beta_{1}=0.5$; $\beta_{2}=-0.3$; $\beta_{3}=0.7$   $F_{j}$  is the first-stage reduced form F-statistic for $x_{j},j=1,2,3$; $F_{1|2,3}$, $F_{2|1,3}$  and $F_{3|1,2}$  are the conditional F-statistics as in (15); $\tau_{\min}$ is the Cragg–Donald minimum eigenvalue statistic; rel bias is the relative bias of the 2SLS estimator, relative to that of the OLS estimator; SY rej freq uses the 5% Stock–Yogo critical values for a maximum 10% total relative bias.

It is clear from the conditional F-statistics that the near rank reduction is due to parameters in the reduced form equations for $x_{1}$ and $x_{2}$. From a straightforward extension of the analytical results for the two-variable case in the Appendix we get that β^3,2SLS is consistent as $\delta_{3}=0$. This is confirmed by the simulation results. The total relative bias in this case is equal to 8.8%, which is less than 10%. It is clear that the conditional F-statistics now provide important additional information to that provided by the Cragg–Donald statistic.

## 5 Conclusions

We have shown that a conditional first-stage F-test statistic can be informative about the information that instruments provide for models with multiple endogenous variables. The conditional F-test is similar to the one proposed by Angrist and Pischke (2009), but takes the variance of the multiple equations into account for testing a rank reduction of one of the matrix of reduced from parameters. Our weak instrument asymptotics is defined as local to a rank reduction of one of this matrix. We find that the conditional F-statistics in a two-endogenous variables model provide the same information as the Cragg–Donald test statistic for testing a rank reduction of one, unless the rank reduction is due to the fact that the instruments are uninformative for one of the endogenous variables. The conditional F-statistics are informative for total relative bias and Wald test size distortions for individual structural parameters. With more than two endogenous variables, the conditional F-statistics can provide additional information regarding the strength of the instruments for the different reduced forms. We therefore recommend in applied work that researchers report standard first-stage F-statistics, the Cragg–Donald statistic and the conditional F-statistics in order to gauge the nature of the weak instrument problem, if any. The Stock and Yogo (2005) weak instrument critical values can be used for the Cragg–Donald and conditional F-statistics. When reduced form errors are conditionally heteroskedastic and/or serially correlated, robust conditional F-statistics can be computed and used as tests for underidentification. However, the exact link of the Stock and Yogo (2005) critical values with the magnitude of the relative bias and Wald-test size distortions no longer holds for the robust statistics and is therefore an important avenue for future research.

## Appendix

### A.1 Cragg–Donald statistic

The Cragg–Donald statistic in the two-variable model is obtained as

τmin=minH(δ,π2)=((π^1π^2)−(δπ2π2))′(Σ^−1⊗Z′Z)((π^1π^2)−(δπ2π2)).

The first-order condition is given by, writing Σ^−1=(σ^11σ^12σ^12σ^22),

−12∂H(δ,π2)∂π2=((δσ^11+σ^12δσ^12+σ^22)⊗Z′Z)((π^1π^2)−(δπ2π2))=0,

resulting in

(δσ^11+σ^12)Z′Zπ^1+(δσ^12+σ^22)Z′Zπ^2=δ(δσ^11+σ^12)Z′Zπ2+(δσ^12+σ^22)Z′Zπ2.

Hence,

π¯2=(δ¯σ^11+σ^12)π^1+(δ¯σ^12+σ^22)π^2δ¯(δ¯σ^11+σ^12)+(δ¯σ^12+σ^22)=(δ¯σ^22−σ^12)π^1+(σ^12−δ¯σ^12)π^2σ^12+δ¯2σ^22−2δ¯σ^12,

and

π^2−π¯2=−(δ¯σ^22−σ^12)σ^12+δ¯2σ^22−2δ¯σ^12(π^1−δ¯π^2);π^1−δ¯π¯2=(σ^12−δ¯σ^12)σ^12+δ¯2σ^22−2δ¯σ^12(π^1−δ¯π^2).

As

((σ^12−δ¯σ^12)−(δ¯σ^22−σ^12))′Σ^−1((σ^12−δ¯σ^12)−(δ¯σ^22−σ^12))=(1−δ¯)Σ^(1−δ¯)=σ^12+δ¯2σ^22−2δ¯σ^12.

it follows that

H(δ¯,π¯2)=(π^1−δ¯π^2)′Z′Z(π^1−δ¯π^2)σ^12+δ¯2σ^22−2δ¯σ^12.

### A.2 Total relative bias

Equivalently to (11) we can write

$$x_{2}=x_{1}\delta^{*}+Z_{2}\left(\pi_{22}-\pi_{12}\delta^{*}\right)+v_{2}-\delta^{*}v_{1}$$

where $\delta^{*}=\pi_{21}/\pi_{11}=\delta^{-1}$. Hence, under LRR1 weak instrument asymptotics, we have

$$x_{2}=x_{1}\delta^{*}-Z_{2}c\delta^{*}/\sqrt{n}+v_{2}-\delta^{*}v_{1}\text{.}$$

As

(1nZ2′Mx^1u1nZ2′Mx^1(v2−δ∗v1))⟶d(ψZ2∗∗uψZ2∗∗(v2−δ∗v1))=N(0,Ω∗⊗Q∗),Ω∗=(σu2σu2−δ∗σu1σu2−δ∗σu1σ22+δ∗2σ12−2δ∗σ12);Z2∗∗=Mx^1Z2;Q∗=plim(n−1Z2∗∗′Z2∗∗)Q∗=plim1nZ2′Mx^1Z2=plim(1n(Z2′Z2−Z2′δx2(δx2′Z(Z′Z)Z′δx2)δx2′Z2))=Q.

It follows that $\psi_{Z_{2}^{**}u}=\psi_{Z_{2}^{*}u}$  and $\psi_{Z_{2}^{**}\left(v_{2}-\delta^{*}v_{1}\right)}=\psi_{Z_{2}^{*}\left(v_{2}-\delta^{*}v_{1}\right)}=-\frac{1}{\delta }\psi_{Z_{2}^{*}\left(v_{1}-\delta v_{2}\right)}$, as e.g. 

Z2′Mx^1u=Z2′u−Z2′x1(x1′PZx1)−1x1′PZu=Z2′u−Z2′x1(x1′Z(Z′Z)−1Z′x1)x1′Z(Z′Z)−1Z′u.

Further,

$$\mathrm{plim}\left(n^{-1}Z^{\prime }x_{1}\right)=\delta \mathrm{plim}\left(Z^{\prime }x_{2}\right),{\overset{˜}{\lambda }}^{*}=\sigma_{v_{2}-\delta^{*}v_{1}}^{-1}Qc\delta^{*}={\left(\delta^{*}\sigma_{v_{1}-\delta v_{2}}\right)}^{-1}Qc\delta^{*}=\overset{˜}{\lambda }\text{,}$$

so we get that

β^2,SLS−β2⟶d−δσuσv1−δv2(λ˜+z˜v)′z˜u(λ˜+z˜v)′(λ˜+z˜v)

and hence, asymptotically,

E[β^2,SLS]−β2=−δE[β^1,2SLS]−β1.

Using this, we can express the total relative bias (4) as

$$B^{2}=b^{2}\frac{\Sigma_{Xu}^{\prime }\Sigma_{X}^{-1/2}\Sigma_{X}^{1/2}D\Sigma_{X}D\Sigma_{X}^{1/2}\Sigma_{X}^{-1/2}\Sigma_{Xu}}{\Sigma_{Xu}^{\prime }\Sigma_{X}^{-1}\Sigma_{Xu}}$$

where

$$b=E\left[\frac{{\left(\overset{˜}{\lambda }+{\overset{˜}{z}}_{v}\right)}^{\prime }{\overset{˜}{z}}_{v}}{{\left(\overset{˜}{\lambda }+{\overset{˜}{z}}_{v}\right)}^{\prime }\left(\overset{˜}{\lambda }+{\overset{˜}{z}}_{v}\right)}\right]\text{;}\;\Sigma_{Xu}=\left(\begin{array}{c} \sigma_{u1}\\ \sigma_{u2} \end{array}\right)\text{;}\;D=\frac{1}{\sigma_{1}^{2}+\delta^{2}\sigma_{2}^{2}-2\delta \sigma_{12}}\left(\begin{array}{cc} 1 & -\delta \\ -\delta & \delta^{2} \end{array}\right)\text{.}$$

Hence

$$B^{2}\leq b^{2}\max\mathrm{eval}\left(\overset{1/2}{\underset{X}{\Sigma }}D\Sigma_{X}D\Sigma_{X}^{1/2}\right)\text{.}$$

As $\Sigma_{X}^{1/2}D\Sigma_{X}D\Sigma_{X}^{1/2}$ is a symmetric idempotent matrix, we get that $B^{2}\leq b^{2}$. To show this, note that

$$\Sigma_{X}=\mathrm{plim}\frac{1}{n}\left(\begin{array}{cc} x_{1}^{\prime }x_{1} & x_{1}^{\prime }x_{2}\\ x_{1}^{\prime }x_{2} & x_{2}^{\prime }x_{2} \end{array}\right)=\pi_{2}^{\prime }Q_{ZZ}\pi_{2}\left(\begin{array}{cc} \delta^{2} & \delta \\ \delta & 1 \end{array}\right)+\Sigma_{V}$$

and hence $\Sigma_{X}D=\Sigma_{V}D$. Let

$$d=\frac{1}{\sqrt{\sigma_{1}^{2}+\delta^{2}\sigma_{2}^{2}-2\delta \sigma_{12}}}\left(\begin{array}{c} 1\\ -\delta \end{array}\right)$$

so that $D=dd^{\prime }$, then $d^{\prime }\Sigma_{V}d=1$ and hence

$$\Sigma_{X}D\Sigma_{X}D=\Sigma_{V}D\Sigma_{V}D=\Sigma_{V}dd^{\prime }\Sigma_{V}dd^{\prime }=\Sigma_{V}dd^{\prime }=\Sigma_{V}D=\Sigma_{X}D$$

and therefore

$$\Sigma_{X}^{1/2}D\Sigma_{X}D\Sigma_{X}^{1/2}\Sigma_{X}^{1/2}D\Sigma_{X}D\Sigma_{X}^{1/2}=\Sigma_{X}^{1/2}D\Sigma_{X}D\Sigma_{X}D\Sigma_{X}D\Sigma_{X}^{1/2}=\Sigma_{X}^{1/2}D\Sigma_{X}D\Sigma_{X}^{1/2}\text{.}$$

### A.3 Stata code

Simple Stata code to calculate the conditional F-statistic $F_{{}_{1}{|}_{2,3}}$ for the case of 3 endogenous variables, x1, x2 and x3, with 5 instruments z1,…,z5 and 2 other exogenous variables w1 and w2 is as follows:

ivregress 2sls x1 (x2 x3 = z1 z2 z3 z4 z5) w1 w2

predict res123, r

reg res123 z1 z2 z3 z4 z5 w1 w2

test z1 z2 z3 z4 z5

scalar Fsw = r(F)*r(df)/(r(df)-2)

di Fsw

## Footnotes

1 The one-variable model as described above was y=Mx^2x1β1+ξ. and so one could ask the question whether the weak instrument relative bias could apply to the OLS estimator in this model instead. The OLS estimator is given by

β˜1,OLS=x1′Mx^2yx1′Mx^2x1=β1+β2x1′Mx^2x2+x1′Mx^2ux1′Mx^2x1

and therefore

$$\mathrm{plim}\;{\overset{˜}{\beta }}_{1,OLS}-\beta_{1}=\frac{\beta_{2}\sigma_{12}+\sigma_{u1}}{\sigma_{1}^{2}}$$

and hence, again

B˜n,1=|E[β^1,2SLS]−β1||E[β˜1,OLS]−β1|≠|E[(λ˜+z˜v)′z˜v(λ˜+z˜v)′(λ˜+z˜v)]|.
